# Stakeholders’ perceptions on the role of professional sports clubs in local community health promotion

**DOI:** 10.1093/heapro/daaf076

**Published:** 2025-06-10

**Authors:** Jack Brazier, Joey Murphy, Charlie Foster, Nick Townsend

**Affiliations:** Centre for Exercise, Nutrition and Health Sciences, School for Policy Studies, University of Bristol, Bristol BS8 1TZ, United Kingdom; Centre for Exercise, Nutrition and Health Sciences, School for Policy Studies, University of Bristol, Bristol BS8 1TZ, United Kingdom; Population Health Sciences, Bristol Medical School, University of Bristol, Bristol BS8 2PL, United Kingdom; Centre for Exercise, Nutrition and Health Sciences, School for Policy Studies, University of Bristol, Bristol BS8 1TZ, United Kingdom

**Keywords:** professional sport clubs, health promotion, intersectoral, physical activity, communities

## Abstract

Charitable arms of professional sports clubs and organizations (PSCOs) offer a range of health promotion (HP) programmes within communities, yet little is known about their role within approaches to HP, particularly from the view of key intersectoral partners. Our study explored the perceptions of the role of PSCOs within local approaches to HP from the perspective of multisectoral stakeholders in a southwest region of England. A qualitative single case study approach was implemented, undertaking semi-structured interviews (*n* = 23) with intersectoral stakeholders spanning the sport, public, voluntary, and health sectors. Findings suggest PSCOs were viewed as important organizations for provision of local HP due to their unique assets, such as stadia, branding, coaching staff and their presence within communities. However, their aims and objectives were unclear to stakeholders and often perceived as motivated by ‘brand drivers’ of the elite club, despite holding independent charitable status. Moreover, stakeholders were generally unaware of evaluation materials created by PSCOs and favoured the development of a co-produced evaluation framework for PSCOs. In conclusion, PSCOs should utilize existing community forums, networks, and working groups to better communicate organizational structure, aims, and provision amongst prospective partners. Better understanding of PSCOs structures and aims would support understanding of organizational readiness and requirements for future collaboration in intersectoral approaches to local HP. Moreover, local policymakers should consider how mutually beneficial partnerships with PSCOs could be formed, and how the unique assets, and reach, of PSCOs can be best utilized within intersectoral approaches to local HP.

Contribution to Health Promotion

- This research offers novel insight regarding the role of PSCOs in intersectoral approaches to local HP.
- Findings suggest that the breadth of PSCOs’ HP projects is largely unknown by local stakeholders; however, their reach and presence within communities are considered to be highly valuable assets to local HP.
- However, PSCOs affiliation with elite sports club simultaneously raise significant concerns regarding the motives and drivers of HP projects.
- To maximize the role of PSCOs for local HP, greater communication and collaboration regarding financial and governance structures of PSCOs is required, alongside increased investment in evaluation and subsequent dissemination of HP projects.

## INTRODUCTION

Within the United Kingdom (UK), national and local long-term plans for health improvement should prioritize primary prevention of non-communicable diseases, focusing on smoking, poor diet, obesity, physical inactivity and excess alcohol consumption (Steel *et al*. 2018). Improving such lifestyle behaviours are similarly a priority for local authorities, who hold responsibility for local public health promotion (HP) in England, in addition to their existing responsibilities for services such as housing, education, and transport (Hunter *et al*. 2022, Local Government Association 2022). Further developing local infrastructure for health, in 2019 the National Health Service (NHS) launched their Long-Term Plan, which set out to embed a systems approach to improve the health of the nation, where ‘integrated care systems’ (ICSs) were established across geographical regions in England (NHS 2019a). ICSs are intended to implement intersectoral approaches to promote and improve the health of regions in which they operate (NHS 2019b). Intersectoral approaches to health are recognized as partnerships between organizations placed within different sectors of society, enabling the sharing of information, expertise, resources, and skills to address health issues and improve health outcomes; often resulting in a process that brings together communities, local authorities, and health, education, sport, and voluntary sectors, and is more sustainable, effective, and efficient compared to if the health sector acted alone (Koelen *et al*. 2008, Bagnall *et al*. 2019, Stansfield *et al*. 2021, Nobles *et al*. 2022).

The charitable arms of professional sport clubs and organizations (PSCOs) have previously been identified as effective settings for, and providers of, HP projects, particularly for weight management and mental health (Wyke *et al*. 2015, Curran *et al*. 2017, Gray *et al*. 2018, Wilcock and Smith 2019, George *et al*. 2022). A key example of effectiveness and acceptability of HP projects delivered by PSCOs can be evidenced through the Football Fans In Training programme, which has since been adopted, adapted and implemented globally (Wyke *et al*. 2015, Blunt *et al*. 2017, Gray *et al*. 2018, Hunt *et al*. 2020, Kwasnicka *et al*. 2020, Maddison *et al*. 2020, McDonald *et al*. 2023). Whilst evaluations of other PSCOs’ interventions have demonstrated PSCOs’ role within HP more widely; particularly amongst men and those at risk of future ill-health (Pringle *et al*. 2013, Parnell *et al*. 2015, Rutherford *et al*. 2021, Timm *et al*. 2025). Moreover, across the UK and Europe, previous research has mapped the breadth of HP programmes delivered within the day-to-day practice (i.e. where academic evaluations have not been conducted) of PSCOs, identifying tailored projects spanning health, education and sport (Lozano-Sufrategui *et al*. 2020, Pringle *et al*. 2021, Røynesdal *et al*. 2021, Brazier *et al*. 2024). Therefore, PSCOs’ HP efforts may be considered as either ‘health promotion through sport’ or ‘health promotion in sport’, whereby sport is used either as a means, or a setting for the delivery of interventions or projects, that sought to improve health outcomes and lifestyle habits of participants respectively (Geidne and Van Hoye 2021).

Whilst PSCOs deliver a breadth of programmes, and have considerable reach within local communities, demonstrating the impact and outcomes of their work is a significant challenge and likely contributes to their exclusion from public health and physical activity policies (Lozano-Sufrategui *et al*. 2020, Pringle *et al*. 2021, Brazier *et al*. 2024). Previous research indicates that both PSCOs and community members view PSCOs as community anchor organizations that have responsibilities to promote health and wellbeing locally (Alonso and O'Shea 2012, Sanders *et al*. 2021). However, local stakeholders’ (i.e. intersectoral actors and decision/policy-makers) perceptions of the role of PSCOs within intersectoral approaches to local HP are unknown. Whilst the need to explore the role of PSCOs in strategic approaches to local HP has been longstanding, there is little evidence to draw upon, particularly from the perspective of local stakeholders (Lansley and Parnell 2016). Moreover, due to funding and resource cuts within the health sector, it is argued that the health sector is likely to become increasingly more dependent on the community sport/PA sector in supporting it achieving its objectives (Duffell *et al*. 2023). However, data suggest that PSCOs are currently somewhat siloed within intersectoral approaches to HP, despite claims that they may be important partners in addressing national health priorities (Lansley and Parnell 2016, Duffell *et al*. 2023, Brazier *et al*. 2025).

Therefore, our study aimed to investigate key stakeholders’ perceptions of PSCOs in an intersectoral approach to HP within BNSSG. To address this aim, the following research objectives were set:

- To investigate key stakeholders’ perceptions of the current and potential role of PSCOs in delivering health and wellbeing agendas.
- To explore the nature of the relationships between PSCOs and partnering organizations in delivering local health and wellbeing promotion programmes in communities.
- To capture and understand the attitudes, perceptions, and values held by key stakeholders around monitoring and evaluation practices on health and wellbeing programmes delivered by PSCOs.
- To utilize key stakeholders’ views to inform future monitoring and evaluation practices on health and wellbeing programmes delivered by PSCOs.

## MATERIALS AND METHODS

To address our research aim, a single qualitative case study design was adopted (Yin 2009). This approach was deemed the most appropriate given its acceptability in exploring real-world phenomena and the meaning individuals associate with such phenomena through their experiences (Yin 2009, Denzin and Lincoln 2011).

**Figure 1.**
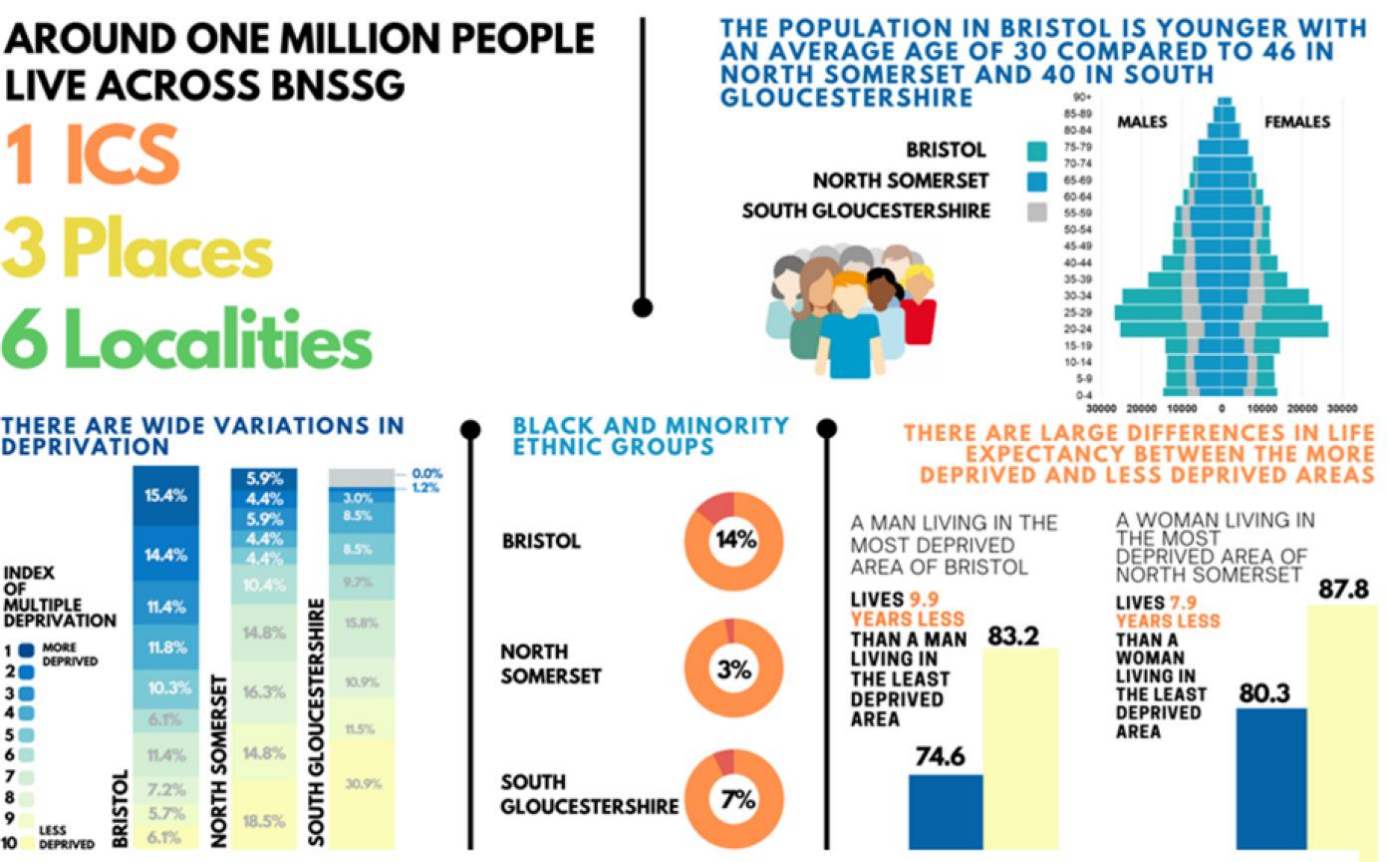
BNSSG ICS population. (BNSSG Healthier Together 2022).

This research was conducted in Bristol, North Somerset, and South Gloucestershire (BNSSG), a southwest region of the UK, where a diverse population of over a million people live in urban, rural, and coastal communities across varying levels of deprivation (see Fig. 1). It is recognized that a preventative approach to ill-health is required within BNSSG, whereby five workstreams have been set up to guide the implementation of the ICS’s strategy: (i) tackling systemic inequalities, (ii) strengthening the building blocks of health, (iii) prevention and early intervention, (iv) creating healthy behaviours, (v) strategic prioritization of key conditions (BNSSG Healthier Together 2023).

Within BNSSG, five PSCOs operate and often adopt football, rugby, cricket, or multi-sport models to deliver HP projects for children, adults, and older adults. All PSCOs within the region hold independent charitable status from their associated elite sport club, but will utilize club stadia to deliver projects, alongside community venues, sport and leisure facilities, and schools. Typical examples of projects include, but are not limited to, weight management initiatives, mental HP, physical activity promotion, inclusion and disability sport, and physical education provision (Bristol Bears Foundation 2025, Bristol Rovers Community Trust 2025, Bristol Sport Foundation 2025b, Robins Foundation 2025). Between the five PSCOs, stadium capacities are reported at 27 000, 11 000, and 8000, demonstrating their ability to promote HP projects and opportunities to those in attendance of fixtures alone. Moreover, engagement with communities outside of matchdays is frequently undertaken, whereby one PSCO reports partnerships with over 40 schools throughout BNSSG, whilst another reports reaching over 8000 people between the ages of 5 and 104 years (Bristol Bears Community Foundation 2025, Bristol Sport Foundation 2025a). The BNSSG ICS have recently set strategic aims of developing a more cohesive and collaborative approach to local HP, in efforts to improve the health of the population and reduce health inequalities (BNSSG Healthier Together 2022, BNSSG Healthier Together 2023). Similarly, local sport and physical activity strategies recognizes the need for improved collaboration and effective partnership working to support physical activity promotion (Bristol City Council 2020, North Somerset Council 2023, South Gloucestershire Council 2024).

### Theory

Our research was conducted from a critical realist philosophical position which proposes that whilst reality exists beyond the observable human world, individual experiences, culture, interactions, and societal structures impact perceptions of reality (Archer *et al*. 2013). Moreover, critical realism lends itself to the exploration of how organizational structures, cultures, and networks can influence human reality (Gorski 2013). This study formed part of a wider project in which a social network analysis was undertaken with organizations in the sample (Brazier *et al*. 2025). It must therefore be acknowledged that existing or previous organizational and personal relationships within the research setting and organizational cultures could influence the perceptions of participants.

### Recruitment

A purposive sampling method was used to invite stakeholders to participate in the study, allowing for the careful selection of participants with specific knowledge and experience related to the research (Kelly 2010). An initial email was sent to members of a local steering group for a PSCO Sport England-funded programme that J.B. and N.T. were involved in. Following this, a snowballing technique was used for further recruitment, whereby those who took part in the interviews signposted the researchers to other potential participants within BNSSG (Knoke and Yang 2008). All participants were initially emailed a summary of the study and an invitation to express their interest in taking part, followed by an information sheet, an infographic summary of the project, and a consent form. All participants were required to provide written consent prior to participation, and verbal consent prior to starting the audio recording of interviews.

### Participants

Participants must have been employed by an organization located within the BNSSG geographic boundary. A total of 65 organizations operating within BNSSG were invited to participate in the study, covering PSCOs (*n* = 5), and public (*n* = 9), education (*n* = 3), health (*n* = 5), sport (*n* = 13), Voluntary Community, Faith and Social Enterprise (*n* = 10) organizations, and primary care networks (PCNs) (*n* = 20) (Brazier *et al*. 2025).

### Data collection

Semi-structured interviews were conducted to gather insight around the perceptions, beliefs, and experiences of key stakeholders involved with intersectoral approaches to local HP within BNSSG (Smith and Sparkes 2018, Roulston and Halpin 2022). The topic guide was developed between J.B., J.M., and N.T., and informed by previous findings and projects undertaken by all authors (see Supplementary File S1).

### Data analysis

All interviews were conducted and audio recorded by J.B. and transcribed automatically via Microsoft Teams. All transcriptions were checked for accuracy against the original recordings by J.B. and then imported into NVivo 12 for analysis. To ensure anonymity of participants, all identifiable information was removed from transcripts and all participants were given a unique ID code. An inductive approach to thematic analysis was taken, whereby Braun and colleagues’ (2016) framework for thematic analysis within sport and exercise research was used to guide the analysis. This process has been reported within Table 1. Moreover, the most illustrative data extracts were embedded within the main text, and further supporting data were placed within a Meaning Unit Table to support higher order and sub-themes (see Supplementary File S2).

**Table 1.** Stages of thematic analysis (informed by Braun *et al*. 2016).

Stage of thematic analysis	Authors involved	Process undertaken
(i) Familiarization with data	J.B.	Familiarization with data was undertaken by J.B. as lead author, this was achieved through both conducting the interviews and accuracy checking of transcripts. Within this process, initial (ii) coding was carried out whereby key points of interest, ideas, and concepts related the research objectives were identified
(ii) Coding	J.B.	Building upon initial coding, J.B. generated semantic labels against data, capturing the explicit meaning of data in relation to the research aim and objectives (Braun *et al*. 2016). Two waves of coding were undertaken prior to the next phase of (iii) thematic development
(iii) Thematic development	J.B., J.M., N.T.	Codes were organized into themes to explore ‘higher level’ pattens with the data. Within this phase, J.M. and N.T. checked the codes developed by J.B. to ensure agreement with the development of initial themes.
(iv) Refinement of themes	J.B., J.M., N.T.	Coded data against themes were checked, and specific consideration was given to the relationships and connection between each theme, and their relation to the research objectives. Consequently, some data were removed as they were more pertinent to other aspects of the wider research project. In addition to this, sub-themes were also developed for the second and third theme reported
(v) Naming of themes	J.B., J.M., N.T., peer-reviewers	The (v) naming of themes was then undertaken initially by J.B. with J.M. and N.T. contributing to the review and refinement of these. Themes were then further refined following peer-review.
(vi) Writing up of results	J.B., J.M., N.T.	Data extracts were used illustratively to offer examples of the analytic claims. Within this phase of analysis, J.B. developed a draft with supporting data extracts which was reviewed and edited by J.M. and N.T. Applicability and relevancy checks of data extracts were also carried out by J.M. and N.T. As a result, some data extracts were shortened and additional extracts were used.

## RESULTS

Twenty-three participants took part in a combination of online and face-to-face interviews whereby the public, private, and charitable sector were represented by individuals operating locally in strategic, managerial, commissioning, and practitioner positions. Participants represented PSCOs (*n* = 4), sport/PA organizations (*n* = 6), Voluntary, Community, Faith and Social Enterprise organizations (i.e. not for profit) (*n* = 4), PCNs (*n* = 4), health sector organizations (*n* = 3), and local government (*n* = 2). It is important to highlight that PSCOs have been categorized separately from sport/PA and social enterprise organizations. Whilst PSCOs could be categorized as such organizations given their core delivery activities and independent charitable status, given the scope of this study we propose their individual categorization is important. Interviews were conducted between January 2024 and May 2024, and ranged between 22.4 and 66.24 minutes in duration.

Four higher order themes and six sub-themes were generated through analysis, and are briefly described within Table 2.

**Table 2.** High level summary of themes.

Theme	Subtheme	Definition
The role of PSCOs in intersectoral approaches to local HP		Stakeholders reported varied awareness of PSCOs HP activity within the region, but suggest PSCOs should have an involvement in local strategic approaches to HP
Key stakeholders’ perceptions of PSCOs	Viewed as a business	This subtheme highlights stakeholders’ perceptions that PSCOs are run as businesses, rather than charities, whereby a focus appeared to be on income generation.
	Viewed as part of the elite club	Stakeholders and PSCOs suggested that the affiliation with wealthy sports clubs raised concerns regarding perceptions of organizational motivators, funding arrangements, and governance.
What PSCOs can offer and the challenges they experience	Assets	Stakeholders and PSCOs identified unique assets or resources that PSCOs hold which may be beneficial to local intersectoral approaches to HP
	Challenges PSCOs face (i) Branding	PSCOs reported that whilst simultaneously beneficial, the tie to elite sports club can hinder their ability to form strategic partnerships.
	(ii) Monitoring and evaluation of HP projects	Whilst the challenge of capturing robust evidence demonstrating the impact of HP projects limits PSCOs ability to foster new collaborations.
Demonstrating the impact of PSCOs	Unaware of PSCOs outputs	Many stakeholders reported little awareness of PSCOs’ evaluation materials and outputs. A challenge similarly recognized by PSCOs.
	How can monitoring and evaluation be improved	A series of practical recommendations for future practice and research concerning the evaluation of PSCOs HP projects

### Role of PSCOs in intersectoral approaches to local health promotion

Stakeholders were aware of local PSCOs projects delivered across a breadth of areas, such as physical activity, sport promotion, and disability sport, specifically around football for visually impaired groups, people living with Down syndrome, and cerebral palsy. Additionally, further references to PSCOs’ projects seeking to support weight management, such as the Man vs. Fat and FIT FANs initiatives were frequently made. Whilst awareness of tailored projects amongst specific populations such as girls football, walking sports, and summer multisport camps was also indicated. Moreover, participants also reported awareness of educational work delivered by multiple PSCOs locally, especially in underserved communities. However, awareness of projects delivered beyond PA and sport, and mental health, were dependent on stakeholders having current or previous relationships with PSCOs and therefore developing awareness of the breadth of their work. Typically, such relationships involved collaboration in project delivery, or the commissioning of PSCOs to deliver HP projects. Where relationships with PSCOs had not previously existed, stakeholders’ perceptions were that PSCOs generally provide opportunities to increase participation in their respective sports.

I think you'd probably look at those organizations, and just think, ‘they’re an elite football team that have the players that are paid, professional coaching staff’. You probably wouldn't realise the work they do in schools, in local communities, and [with] health conditions.—Sport and Physical Activity Body, Sport/PA Sector

In contrast to this, those representing PSCOs suggested that PA and sport promotion was only one area of their focus, and often workstreams focusing on education, older adults’ wellbeing, and school-based initiatives were given equal resource (see Supplementary File S2). At a strategic level, stakeholders reported PSCOs could be key in supporting statutory organizations, such as local governments and ICS, in achieving system-level goals. Specifically, PSCOs were identified as potential partners who could be pivotal in reducing health inequalities through their delivery of HP projects and ability to engage communities that statutory organizations often struggle to engage with (see Supplementary File S2).

I think probably the most important bit, that we collectively need to shout about more, is the fact that they are embedded in those communities. And the power of allegiance for people in our communities to that sports club is something that we need to probably play on more. Because they (PSCOs) can reach individuals in a way that clinical healthcare services can't.—Active Partnership—Sport/PA Sector

However, participants suggested that a greater collaborative approach is required to achieve this, whereby strategic joined-up thinking, organizations harnessing relationships, and embedding themselves within communities, is essential (see Supplementary File S2). Moreover, local stakeholders believed that PSCOs should be involved in such strategic approaches, suggesting they are likely to be fundamental in supporting the ICS to achieve its objective of improving population health (see Supplementary File S2). Participants highlighted that greater understanding of the aims, objectives and governance structures of PSCOs is required to identify shared priorities and objectives; in efforts to develop more effective partnerships within intersectoral approaches to local HP (see Supplementary File S2).

### Key stakeholders’ perceptions of PSCOs

#### Viewed as a business

A key theme interpreted from the data was how PSCOs are perceived as organizations by stakeholders across BNSSG. Generally, stakeholders reported that they believed PSCOs play a significant role in providing HP opportunities locally, yet reservations exist regarding the aims and objectives of PSCOs (see Supplementary File S2). PSCOs were often viewed as being run as businesses, rather than charities (see Supplementary File S2). Whilst it was recognized by stakeholders that as a charity PSCOs need to generate revenue to deliver projects, cover overheads, and pay staff, some participants perceived PSCOs to be more interested in potential financial benefit or the receipt of funding from collaboration. In turn, this raised concerns amongst some stakeholders, who suggested this portrayed PSCOs to be commercially driven and subsequently left stakeholders feeling cautious about future collaboration. The commercial perception of PSCOs was also highlighted by participants representing PSCOs, whereby it was suggested that PSCOs need to communicate the charitable focus of their work better, demonstrating their independence from the elite sport club (see Supplementary File S2).

I see them as a voluntary sector or social enterprise organization that obviously have got some, you know, charitable or sort of, I suppose socially enhancing drivers, but I think they've also, if I'm honest, I think they've got some brand drivers.—SC, Voluntary, Community, Faith and Social Enterprise Sector

#### Viewed as a part of the elite club

Contributing to the perception of PSCOs run as commercial enterprises, stakeholders viewed PSCOs as a part of the elite sport club, rather than an independent charity of the elite club (see Supplementary File S2). The lack of clear distinction between the elite sport club and the charitable entity of clubs was as a further concern of stakeholders and acted as a barrier to collaboration. Specifically, stakeholders reported PSCOs often voice concerns regarding lack of financial resource and the need to agree to funding arrangements ahead of committing to collaboration, given their independence from the elite sport club and subsequent need for independent fundraising. This was questioned by some stakeholders due to the assumption that PSCOs would, or should, receive adequate funding from the elite sport club, given the well-documented level of financial resource that is available for player salaries and stadium costs (see Supplementary File S2). Moreover, due to perceived relationships between the elite sport club and the PSCO, projects and initiatives delivered by PSCOs were viewed as being partially motivated by brand enhancement, ticket and merchandise sales, and often as a form of corporate social responsibility (see Supplementary File S2). Ultimately, raising concerns regarding the genuineness of some PSCO activities.

Participants representing PSCOs recognized they were typically perceived as being intrinsically linked with the elite sport club, rather than an independent organization (see Supplementary File S2). PSCO participants suggested whilst brand association with the elite sport club is a key asset to their HP work, the association can also lead to this misconception. PSCOs suggested that greater work was required, and relationships needed to be developed across BNSSG to emphasize the charitable status and independence of PSCOs.

We're an independent organization, we have a board of five trustees that are independent. I'm not employed by the [elite club]. I'm employed by [the community trust], which is a separate charity. However, the man in the street, or the funder in the street, probably thinks [the community trust] are looking for money, they’re owned by the [elite club], the [elite club] owners are wealthy. Why, either why aren't they paying for this?—PSCO2

### What PSCOs can offer and the challenges they experience

#### Assets

Whilst the association to an elite sport club can raise concerns, participants reported the association is simultaneously a unique and beneficial asset that PSCOs hold. It was suggested that association with the badge and brand of an elite club brings ‘prestige’, ‘credibility’ and ‘kudos’ to PSCOs’ HP projects and initiatives. Moreover, brand association was also highlighted by stakeholders as an asset that could be potentially effective in engaging with communities that statutory and healthcare organizations struggle to engage with. Moreover, the embeddedness of PSCOs within communities, and the fan base of elite sport clubs, were reported as key assets that PSCOs can utilize to engage with community members and support intersectoral approaches to local HP. Participants reported that not only does such reach facilitate PSCOs to engage with high volumes of individuals via their programmes, it could also provide partner organizations with the ability to promote and provide wider HP opportunities (see Supplementary File S2).

I think there are a lot of things [PSCOs offer]. I mean, they have the kudos of the communities that they live in and work in, and you know, that for disadvantaged people, particularly children, can have huge power.—Local Authority2, Public Sector

Furthermore, PSCOs’ ability to embed specific and/or multi-sport coaches within communities, who are able to develop and nurture relationships across the city, was a particularly attractive asset (see Supplementary File S2). Despite PSCOs’ coaches traditionally holding roles in sport and PA promotion, these findings demonstrate stakeholders’ perceptions that coaches may have a role in wider HP. Moreover, PSCOs’ access to diverse funding streams due to their charitable status was also referenced, highlighting their ability to secure limited funding opportunities from national governing bodies.

#### Challenges PSCOs face

##### Branding

Stakeholders suggested that in some cases the branding of PSCOs may actually be ‘off-putting’ for both community members and potential partnering organizations, due to association with sport, and the magnitude (i.e. money and resources) of elite sport clubs (see Supplementary File S2). Moreover, due to brand association, PSCOs highlighted that it is important to acknowledge that they are limited to operating within the geography that they are based in. PSCO participants suggested that a key component of their ability to recruit and engage with community members, was their ability to utilize peoples’ familiarity and affiliation with the name and brand of the PSCO. Therefore, operating outside of the region was not deemed sensical; in turn, accessing national funding, aside from respective governing bodies (i.e. the English Football League), was considered challenging.

The benefits of our delivery, i.e our branded delivery, are limited. We can't deliver outside of Bristol, you know, we could, but it wouldn't make any sense. So national funding becomes difficult for us to access in that respect… However, if you take away our link to the football club, you then take away what attracts people to what you do. So it's a real catch 22 situation.—PSCO2

##### Monitoring and evaluation of HP projects

A considerable challenge highlighted for PSCOs was their ability to showcase the impact of the projects through sound monitoring and evaluation (M&E) of programmes. Time and resources allocated to M&E, alongside PSCO staffs’ knowledge skillsets concerning M&E were highlighted by PSCOs as the specific challenges in showcasing the impact of their programmes (see Supplementary File S2). Understanding what data to collect, how to collect it, and how to report findings, were identified as key challenges (see Supplementary File S2). Stakeholders suggested that PSCOs were not alone in this challenge, which was perceived as a shared issue across BNSSG.

I think it's a sort of generic challenge across the Foundations, how we best demonstrate the value of our work … Sometimes it's proven difficult to capture even some simple media—to then, to stick in the right place [and] in the right hands to demonstrate our impact.—PSCO3

### Demonstrating the impact of PSCOs

#### Unaware of current outputs

Participants were generally unaware of M&E outputs, such as impact reports, created by PSCOs, including organizations that held strong relationships with them. Where stakeholders did report awareness of these outputs, it was suggested that often data produced and reported was beneficial in understanding the reach of projects; however, more in-depth and sophisticated insight was sought by stakeholders. The reporting of ‘output’ data, such as engagement hours or sessions delivered, was considered insufficient to understand the impact of activities (see Supplementary File S2). Additionally, stakeholders suggested to highlight the impact of their work, but also to communicate the aims and breadth of their activities, PSCOs should better utilize existing local networks, working groups, and communication channels, such as local community forums, PCN meetings, and community-led events (see Supplementary File S2).

We all know that attendance figures doesn't really give you a great deal at all, does it really? Ultimately what was the outcome? You know, what is the impact on people's health and well-being? I think it needs to be more aligned to the types of information that's needed, to really demonstrate that the work they're doing is contributing towards the priorities of the wider system.—Local Authority1, Public Sector

#### How could M&E be improved?

Alongside having specialist M&E staff within PSCOs, it was suggested that support from funding organizations, such as Active Partnerships, would be highly beneficial in developing and improving M&E (see Supplementary File S2). PSCOs reported that national programmes are often easier to demonstrate impact, as funders provide clear aims and objectives for projects, alongside M&E protocols, measurement tools, and reporting frameworks. Conversely, where projects were developed by PSCOs, little support was in place to facilitate more robust M&E, especially where projects were subject to short-term funding.

That was a programme where it [an evaluation framework] was provided, it was very easy and I think it was probably very successful in the sense of showing it. Other projects that we deliver obviously are a lot more difficult, and particularly projects if we develop them ourselves.—PSCO2

Participants expressed support for the idea of developing a common M&E framework for PSCOs, suggesting that such a tool could prove essential for developing how PSCOs can contribute to intersectoral approaches to local HP. Importantly, participants suggested this approach would need to be flexible to accommodate the breadth of PSCOs projects, spanning across topics such as mental health, education, and physical activity, which may be a considerable challenge (see Supplementary File S2). The importance of involving intersectoral stakeholders in the development of such a framework was also strongly supported by PSCOs and stakeholders (see Supplementary File S2).

I think would be really useful. I think you need to look at, you know, for [us], it's health, education, sport and inclusion. Those are our four quarters within that all of our project sit… if everyone was singing from the same hymn sheet, I quite like that idea. It makes things that much [more] measurable.—PSCO2

## DISCUSSION

Our study identified that stakeholders supported the role of PSCOs in intersectoral approaches to local HP, suggesting they have a pivotal role in providing opportunities within local communities. Moreover, stakeholders suggested PSCOs may play an important role in supporting statutory organizations, such as local governments and the NHS, in reaching communities they have previously struggled to engage with. However, concerns regarding PSCOs’ drivers and motivations for delivering HP projects were reported as a potential barrier to the development of further relationships within BNSSG. Furthermore, PSCOs identified that capturing the impact of their work and disseminating such outputs was a significant challenge, in turn, hindering the knowledge and awareness of the breadth of programmes delivered by PSCOs locally. Previous research has explored the development of PSCOs over recent years, alongside the strengths and challenges they face in HP activity, however, their role within intersectoral approaches to HP was not explored, nor were stakeholders’ perceptions of PSCOs (Pringle *et al*. 2021, Sanders *et al*. 2021). To our knowledge, this is the first study that has explored the views and perceptions of multisectoral stakeholders spanning health, local government, not-for-profit, and sport organizations, on the role of PSCOs within intersectoral local approaches to HP.

Over the last 25 years, the diversification of programmes and initiatives delivered by PSCOs has significantly grown, whereby a variety of programmes across health, sport, and education are widely offered; reflecting the role of PSCOs in HP both through, and in, sport (Lozano-Sufrategui *et al*. 2020, Geidne and Van Hoye 2021, Pringle *et al*. 2021, Røynesdal *et al.* 2021, Sanders *et al*. 2021, Brazier *et al*. 2024). However, our findings suggest that at a local level, stakeholders are typically unaware of the breadth of programmes delivered, and generally associate PSCOs’ activities as being related to increased participation numbers in respective sports. Despite this, stakeholders propose that PSCOs are important partners to statutory organizations if they are to address local health priorities, and therefore recognize their capacity to deliver both HP through and in sport (Geidne and Van Hoye 2021). To develop awareness of opportunities delivered by PSCOs, our findings suggest the use of existing local intersectoral infrastructures, such as practitioner networks, practitioner working groups, or community groups, may be highly beneficial for dissemination of opportunities and outputs, and building capacity for collaboration within intersectoral approaches to local HP. Findings reported within a social network analysis of this region suggest that PSCOs further developing their relationships with local governments and the active partnership, who hold responsibility for public health and PA promotion respectively, may be particularly important for building relationships and collaborations (Brazier *et al*. 2025). Moreover, PSCOs relationships with not-for-profit community organizations were similarly lacking, which may be a further area for PSCOs to develop relationships with, given their embeddedness and understanding of local health priorities (Brazier *et al*. 2025). Our findings highlight the lack of understanding of PSCOs’ independent governance and funding structures, which alongside stakeholders’ experiences of previous collaboration with PSCOs, provide useful context and explanation for the limited connectedness of PSCOs within local intersectoral HP approaches (Brazier *et al*. 2025). Whilst these findings are localized, the combined approach of social network analysis and semi-structured interviews offer a replicable model for future research to explore the role of PSCOs in local approaches to HP globally.

Pringle *et al.* (2021) reported from interviews with PSCO staff that many clubs in the UK were in partnership with local stakeholders, such as local governments and Integrated Care Boards, yet this varied by club and locality. Despite these existing relationships with decision/policymakers, PSCOs did not hold a clear position within local health and care strategies (Pringle *et al*. 2021). Our findings suggest stakeholders see a role for PSCOs in strategic approaches to health and care, and in some cases, PSCOs were involved in relevant strategic networks and working groups. However, it must be acknowledged that differences in organizational and sectoral priorities as well as objectives can bring challenges to such collaboration (Misener and Misener 2016). The notion of speaking different ‘languages’ and working towards different agendas and priorities were recognized within our data, particularly from PSCOs. Whilst stakeholders similarly reported concerns regarding the commercial aspect of PSCOs, specifically in relation to their motivators to deliver HP projects. These concerns are likely founded on the well-documented corporate social responsibilities of elite sport clubs, the promotion of unhealthy behaviours often witnessed within sport stadia, and the wealth underpinning professional sport (Brown 2016, François *et al*. 2019, Purves *et al*. 2020, Bostock 2021). However, income generation, through the delivery of HP interventions, is considered a measure of success, and is vital for sustainability of PSCOs and their projects (Pringle *et al*. 2014, 2016, Sanders *et al*. 2021). Our findings suggest further research exploring the relationships between stakeholders and PSCOs is required in order to understand shared priorities and objectives, and to develop and implement strategic intersectoral approaches to local HP. However, research should consider the perspectives of wider sport-related disciplines to ensure that future evidence explores the intersectionality of HP, sport, and disciplines such as sport business or management (Geidne and Van Hoye 2021). Developing research that explores such wider methodologies, theories, and evidence bases, may be particularly important within the context of PSCOs given the nature of concerns reported by stakeholders within our findings. Moreover, findings may inform how intersectoral approaches to local HP could effectively utilize limited resources and assets; in turn, supporting local, national, and international health agendas.

Furthermore, our data suggest the independence of PSCOs as charitable entities from the elite sporting club is muddied, highlighted by both stakeholders and PSCOs. Community Trusts and Foundations are not-for-profit charitable entities with independent organizational structure and strategic visions, which are similarly governed and financed independently from the elite sport club (Sanders *et al*. 2021). Amongst PSCOs and wider community sport organizations, it is important to consider their priorities are often concerned with funding and resourcing that will either sustain or improve basic operational delivery; demonstrating the need to consider how differing priorities within sport settings may influence the motivation to engage in HP activity (Misener and Misener 2016, Geidne and Van Hoye 2021, Sanders *et al*. 2021). Moreover, despite recent claims suggesting there is an increasing interdependence between community sport and health sectors, calls for collaboration from national and/or local policymakers are often made without consideration to community sport organizations’ capacity for collaboration (Misener and Misener 2016, Duffell *et al*. 2023). Forming partnerships between sport settings (i.e. between PSCOs) is similarly challenging, whereby identifying a common goal and organizational resource to support meaningful partnerships are the biggest constraints (Donaldson *et al*. 2021). Social network analysis data within this locality suggest that PSCOs’ partnerships with key stakeholders, and each other, are limited; whilst qualitative findings reported here suggest a lack of clarity regarding PSCOs’ aims, priorities, and structure, are a significant barrier to developing meaningful partnerships that could maximize PSCOs role within local approaches to HP. Therefore, we propose that to enable better collaboration with, and amongst, PSCOs, there is a need for improved methods for effective communication amongst key stakeholders across all sectors, to promote better understanding of organizational aims, structures, and delivery across the region. In turn, generating a better understanding of organizational priorities and capacity for collaboration within this intersectoral approach to HP.

Moreover, literature has consistently highlighted unique assets of PSCOs, such as stadia and club branding, as a crucial concept to programmes, often acting as the initial attraction to participants (Pringle *et al*. 2013, Jones *et al*. 2019, Wilcock *et al*. 2021). Our data suggest that this initial ‘hook’ into communities could be potentially effective in engaging individuals and/or communities that statutory services struggle to engage with, particularly for those community members who may already have an affiliation with a PSCO. Such claims are further supported by evidence that suggests PSCOs are effective in recruiting men from lower socio-economic groups and those at risk of future ill-health (Pringle *et al*. 2013, Timm *et al*. 2025). Specifically, leveraging assets such as PSCO branding and presence within communities and developing gender-sensitized interventions are vital to recruitment, whilst interventions that promote trusting and supportive environments with like-minded men, and offer opportunities for competition and self-measurement of success, support intervention adherence (Timm *et al*. 2025). A key example demonstrating the need for careful planning of PSCOs HP projects can be drawn from the Healthy Stadia project, whereby over 2 years 64 000 participants registered interest in a sport and PA promotion programme designed for inactive low socio-economic communities (McKenna *et al*. 2018). However, below 50% of these participants ‘activated’ attendance by attending the first session, reflecting PSCOs’ potential reach within HP projects, but the lack of support available to aid the development of interventions (McKenna *et al*. 2018). Our findings suggest that further support is required by PSCOs for the delivery of HP projects, specifically for evaluation. Supporting frameworks for HP delivery have been developed in other contexts, namely, the Health Promoting Sports Club Framework, a tool developed to support voluntary sport organizations in the planning, delivery, and evaluation of HP activities (Van Hoye *et al*. 2021, Johnson *et al*. 2024). Of particular interest to this study, it suggests the adoption of dynamic intervention strategies that use individuals’ sense of belonging to a club, alongside the interaction between individuals and the environment, are crucial to successful HP interventions delivered by voluntary sports clubs (Van Hoye *et al*. 2021). Our findings and that of previous literature, suggest that such strategies or components are similarly applicable to PSCOs, whereby capitalizing on concepts such as fandom, or gender-sensitized environments, may have important implications for HP. Furthermore, in comparison to this framework, communications with partners, resources and capacity to develop partnerships, and monitoring and evaluation, are all recognized as key intervention strategies or components (Van Hoye *et al*. 2021, Johnson *et al*. 2024). Our findings equally suggest that similar strategies and intervention components are likely crucial to PSCOs’ involvement in intersectoral HP and further emphasize the need to consider how concepts that fall under broader sport-related disciplines, such as corporate social responsibilities or the motivations of the elite sport club, influence HP activity amongst PSCOs. However, our findings and, further analysis within this region, suggest that PSCOs are somewhat siloed in their HP activity, whereby communication and understanding of PSCOs delivery and aims is a considerable barrier to collaboration (Brazier *et al.* 2025). In addition to our recommendations for improved communication within this locality, we propose it is of interest to public health practitioners, PCNs, and NHS organizations to explore how locality-based relationships with PSCOs could serve as a new pathway for offering services within communities, rather than operating in silos. Matchdays and stadia are often used for health screening, and dissemination of HP opportunities, and within England and Wales 83% of the most deprived neighbourhoods are within a 10-mile radius of an English Football League club (English Football League 2022). Further exploration of the reach and effectiveness of such efforts is required to better understand if PSCOs are effective in engaging with those at most risk of experiencing health inequalities. Moreover, future research may wish to explore further similarities and differences between the Health Promoting Sports Club Framework and PSCOs approach to local HP.

The challenges for M&E amongst PSCOs have been well documented whereby time, resources, and skillsets are significant barriers to more rigorous evaluation (Lozano-Sufrategui *et al*. 2020, Pringle *et al*. 2021). Our findings similarly suggest that PSCOs often lack staff who have experience, skills, and the time to focus upon M&E; however, stakeholders across the locality also suggested that M&E of HP programmes is also a considerable challenge; often due to resource availability and short-term funding constraints. Participants expressed support for a common M&E framework that could be adopted by PSCOs, which may in turn address stakeholders’ concerns regarding the reliability upon ‘output’ data to demonstrate impact. A recent review suggests PSCOs commonly rely upon measures such as engagement figures and anecdotal case studies to demonstrate impact of HP projects (Brazier *et al*. 2024). Specifically, a co-produced, framework that could facilitate a combination of outcome data and case studies across a breadth of outcomes was recommended by stakeholders as a potentially useful tool. Such a tool would support PSCOs to develop more robust evaluations of HP projects, which are often underfunded and under-resourced, whilst also supporting the recognized need for the development of strategic partnerships between PSCOs and multisectoral stakeholders (Pringle *et al*. 2014, Sanders *et al*. 2014, Lansley and Parnell 2016, Pringle *et al*. 2016). However, to achieve this, our findings suggest organizations within intersectoral approaches to local HP need to prioritize first understanding organizational aims and objectives, and how they might align to local, regional and national health priorities. In turn, supporting the identification of specific outcomes and objectives to measure progress against.

### Study limitations

Firstly, our study was conducted at a locality level, and therefore only included participants within BNSSG. Therefore, the insight generated from our study may not be generalisable to other localities, however, the findings provide new knowledge for future practice within BNSSG, and when aligned to the findings of Brazier *et al*. (2025) offer a replicable model for future research within the UK and internationally. Moreover, whilst we attempted to contact all 19 PCNs within BNSSG and organizations within the education sector identified through social network analysis, our sample included limited representation from PCNs (*n* = 4) and no representation from the education providers. Therefore, limiting the insight our research gathered on PCN’s views on the role of PSCOs in local HP. Future research exploring the role of PSCOs in intersectoral approaches to HP may wish to consider utilizing additional methods, such as stakeholder workshops, to improve recruitment across these sectors (Brazier *et al*. 2025). Furthermore, the use of snowball sampling may be considered a limitation within this study, given the risk that only organizations familiar with PSCO HP activity would participate in the study. However, social network analysis data demonstrate that PSCOs were somewhat disconnected within the region, and therefore few participants within this study directly partnered with PSCOs. Subsequently, this approach has supported the development of important findings and recommendations for future practice within this locality, in addition to a replicable model for future research exploring PSCOs integration within intersectoral approaches to local HP.

## CONCLUSION

Our study explored multisectoral stakeholders’ views and perceptions’ of PSCOs within an intersectoral approach to local HP. The findings suggest that in the first instance, stakeholders perceive PSCOs as important actors in local approaches to HP offering opportunities including but not limited to PA promotion, weight management, mental HP, and inclusion sport. Specifically, the unique assets that PSCOs hold, such as ‘the badge’, stadia and bespoke coaching, are attractive to wider stakeholders, and offer a potential avenue for engaging with communities that statutory organizations have previously failed to work with effectively. However, the association of PSCOs to the elite sport club simultaneously acts as a hinderance, whereby the organizational structures, aims, and drivers of PSCOs are unclear to stakeholders. Consequently, stakeholders are concerned that PSCOs’ HP efforts within communities may be partially, or wholly, driven by brand enhancement. Locally, there is a need for future practice to enable greater communication and collaboration to aid better understanding of organizational structures, delivery, resources and aims. Moreover, future research and practice should seek to capture shared priorities and identify opportunities to build partnerships working towards shared local goals and better use of limited resource. Such efforts may wish to build upon the social network analysis undertaken in this region through stakeholder and asset mapping to support the development of a more cohesive and collaborative intersectoral approach to HP. Moreover, we propose that the mixed methods approach utilized within this research project is a replicable model for exploring the role of PSCOs in intersectoral approaches to local HP both nationally and internationally.

## Supplementary Material

daaf076_Supplementary_Data

## Data Availability

The data underlying this article will be shared on reasonable request to the corresponding author.
