# Supplementary material for: Stakeholders’ perceptions on the role of professional sports clubs in local community health promotion
Source: Health Promot Int. 2025 Jun 10;40(3):daaf076. doi: 10.1093/heapro/daaf076 (PMC12150026; doi:10.1093/heapro/daaf076)
Supplement: daaf076_Supplementary_Data [file daaf076_supplementary_data.zip › Supplementary File 1_Topic Guide.docx]

*Topic Guide*

**Q. Do you work with any PSCOs in the region?**

- *If so, how would you describe this relationship?*
- *If not, would you like to? Any in particular? Why?*

**Q. Do you think there are any organisations within the local health and wellbeing system that would particularly benefit from collaborating with PSCOs?**

- *Why? What do you think could be achieved?*

Role and Impact of PSCOs in the Health and Wellbeing of Local Communities?

**Q. Do you think that PSCOs have a role within the local health and wellbeing system?**

- What role do you think they play?
- Is this the same view across all local PSCOs?

**Q. Do you believe that PSCOs in Bristol could positively impact the health and wellbeing of communities?**

- *If yes, in what way – Sport and P.E provision? PA opportunities? Mental health? NCDs (e.g diabetes?) Weight management?*

**Q. Do you think there is anything unique, or special, that PSCOs can offer their local communities through their health and wellbeing programmes and services?**

**Q. Is there anything that you believe could heighten or improve PSCOs role and/or impact within local health and wellbeing systems?**

**Q. Do you believe that PSCOs could, or even should, be formally and clearly recognised within health promotion policies and strategies by policymakers?**

- *Is there work to be done to reach this point?*
- *What makes you say yes/no?*

Monitoring and Evaluation

**Q. What are you views on the current monitoring and evaluation practices of PSCOs?**

*What are your thoughts on commonplace practices such as impact reports and anecdotal case studies?*

*What do you perceive to be the strengths and weaknesses of these?*

*What would your organisation like to see more/less of?*

**Q. When trying to showcase the impact of PSCOs programmes, interventions and services, what data would your organisation find most beneficial?**

*How would you like this to be presented?*

**Q. How do you believe PSCOs could best showcase the potential impact of their programmes on local community health?**

*Is development of current monitoring and evaluation practices required?*

*What do you perceive as the barriers to this?*

*What would this help PSCOs achieve?*

Common Reporting Framework

**Q. What are your views on a common impact reporting framework for PSCOs?**

*- Benefits…What are they? To who?*

*- Challenges…What are they? To who?*

*- Feasibility… Is a common impact reporting framework feasible for PSCOs? (different size PSCOs, staff time to complete, staff time to consult on its development)*

***Q.*** ***Are there any other thoughts you have on a common impact reporting framework for local community health and wellbeing programmes delivered by PSCOs?***
