# Supplementary material for: Stakeholders’ perceptions on the role of professional sports clubs in local community health promotion
Source: Health Promot Int. 2025 Jun 10;40(3):daaf076. doi: 10.1093/heapro/daaf076 (PMC12150026; doi:10.1093/heapro/daaf076)
Supplement: daaf076_Supplementary_Data [file daaf076_supplementary_data.zip › Supplementary File 2_Meaning Unit Table.docx]

| **Theme identified** | **Meaning Unit** |
| --- | --- |
| **Role of PSCOs in local health promotion system** | “We use sport as a tool, or football as a tool, to engage with young people and families who are socially marginalised in areas of deprivation, in either health education or positive activities through youth engagement.” – PSCO4  “Our Extra Time programme that isn't the funded one here we've been doing for 20 odd years, which is our over 60s group. They come here every week for two hours, physical and social activities.” – PSCO2  “We've got a pillar of health and well-being as part of the Foundation deliverables - in terms of outcomes. And [it] sits [in] part of our strategy... to give you some context, education, and employability, older people, disability inclusion, and our community [sport] provision, seem to be the yeah, the most popular, sort of, ‘outputs’, if you like.” – PSCO3  “[Working with PSCOs] is absolutely fundamental in helping them (the ICB) to achieve their outcomes. They can set the strategy, but it's only by working collaboratively and in partnership with these organisations that they can actually deliver and achieve anything.”– AUB1, VCFSE Sector  “I think there's a huge opportunity to further develop and align the work that the sports [community] trusts do, and opportunities to support and connect in different places. There's some work, obviously good work, taking place - it's getting back to that shared purpose and that shared understanding, isn't it?” –Local Authority1, Public Sector  “Organisations such as the charitable trust of the sports clubs support the delivery of various programmes, interventions, and I guess ultimately to support some of the outcomes that we're trying to deliver as a sort of a city.” –Local Authority1, Public Sector  “I think that they're (PSCOs) really key, it feels a bit like everyone's sort of trying to understand and navigate where they fit in within the wider system because it's huge and it takes quite a lot of unpicking. And I think that where they might struggle.” – ICB, Health Sector |
| **Key stakeholders’ perceptions on PSCOs** |  |
| *Viewed as a business* | “They're a huge resource, and they have a have a great reach. But of course I think like I said at times it's been very much, ‘well, yeah, we can do that, but what's in it for us? What can you do for us kind of thing?’.” - Local Authority1, Public Sector  “You know, ‘how do we promote our club and get more fans?’ Because ultimately, there's that part of it, you know, we need to be aware that there's that part of it as well. If the clubs are raising their profile, you probably think that they would want to gain more fans and more people coming to watch their games and buy their merch, and all the rest of it.” – PCN1, Health Sector  “But when, I think some of the perception of [PSCO] is misplaced around how we’re very commercial. We've got a commercial element to our programme that enables to do lots of charitable good, [but]we haven't told that charitable story well enough.” *–* PSCO1 |
| *Viewed as a part of the elite club* | “Yeah, potentially. I think what you have is again, when you're attached to professional club, what the front facing thing you see is the professional club. I think you have to dig a bit deeper to then find out they do all these other things through the Community Trust.” –Sport and Physical Activity Body1, Sport/PA Sector  “It’s just that ultimately the drivers that are set, are set at the top of the organisation and I don't mean the Foundations, I mean at the top of the sports clubs, the professional sports clubs, and ultimately, they are commercial organisations.” ­– SC, VCFSE Sector  “I think it's about 80 odd clubs within [XXX] at the moment, that are mostly a couple of people from each club that are just passionate people doing great things, if they can make things work with a very, very little resources they have - surely the Foundations and Trusts could, could maybe do a bit more with the vast amount of resources that they have?” – Community Sport Organisation 3 – Sport/PA Sector  “I appreciate professional sports clubs are businesses [and] need to balance their books, and have to run quite a tight business model as well. But for some reason I can't help thinking that if you can pay a sports person X-£1000, there’s probably a little bit of money around that could be promoting sports in local schools, or in local communities that have difficulty accessing sport.” – PCN1, Health Sector  “They (PSCOs) appear to have resource, although as you said, they're a charitable organisation, which I think sometimes gets lost because often the perception is that they are attached to a, sort of, a [elite] sports club with a very very wealthy owner, and therefore actually, if we're talking about sort of funding and things - where and who could be providing that?” –Local Authority1, Public Sector  “Because specifically for us as the (PSCO), our issue is that we're connected to a professional football club, so the assumption is made instantly – ‘oh, you guys do a football’. Okay, the actual, if you look across all our sessions, the amount of sessions that are football, where people are playing football, isn't that actually that high.” - PSCO4 |
| **What PSCOs can offer the system and the challenges they experience** |  |
| *Assets* | “If you've got young people activities or children's activities, you feel like you've got a good, you’re buying into the club that perhaps you support, or a professional organisation which is in a high level team, you know, that brings its own clout and prestige, I think.” – Sport/PA Provider1, Private Sector  “Ultimately, you grow up in that area, support that football team or their rugby team and or cricket team… you kind of feel like you've been part of it because you've been to the stadium, you paid your match fees, and there's an ownership of the community.” – Sport/PA Provider1, Private Sector  “It's not like just ‘oh so and so is running a football get together on the weekend’, you know, this is [Bristol] City [Football Club], this is [Bristol] Rovers [Football Club]. This is, or, you know, Bristol [Bears] Rugby. …. That in itself would create buzz and excitement, and is more likely to get people along and doing things even if no professionals showed up.” – PCN1, Health Sector  “Callum Sheedy the rugby player, did a film for YouTube talking about mental health. Now I can't remember exact numbers because it's a few years back now, but I think there were over 10,000 views within a week of his YouTube clip. If some public health professional went on and created something from a local authority, I'd be astonished if they got 100 views within a week.” - SC, VCFSE Sector  “We know the power of our individual sports and the brands that they've got is everything, and can be far more engaging than somebody, you know, sticking a leaflet [up] or from a social prescriber at a local health centre, for example. So yeah, so that's the first one, that is that power. The second one is our reach. We've got thousands, hundreds of thousands of people that we can engage with quickly, again using that brand and the data that we've got in order to introduce, or change, or what's the word I’m looking for, influence in a positive way.” – PSCO3  “They (PSCOs) of course have access to facilities, they have access to coaches, they have access to the (elite) sport club - so as a sport entity in its own right, they're of course really important.” – Active Partnership – Sport/PA Sector  “Look they’re a fantastic resource, aren't they? You know, in terms of the access to staff that they have - they have a large staff team who have a footprint within and across local communities at various different levels…From that sort of operational boots on the ground, sports coaches, to the people who are sort of developing and working on the sort of the educational programmes and sort of, the events that they run etcetera.” *-* Local Authority1, Public Sector |
| *Challenges PSCOs face* |  |
| *Branding* | “By having that kind of sport branding against it, then you know, I can also understand that might put a certain percentage of the population off because they'll perceive themselves as not having a pair of trainers or, you know, not being sporty, or, you know, for whatever reason, they don't necessarily see people that look like them, they see sporty people in track suits. So there's a flipside to it”– Community Sport Organisation 1, Sport/PA Sector  “The brand, the badge, I mean that's a really, really strong pull - for some people, not everybody. I would use the analogy … of the Tesco's moving in on the corner of your road and closing down the small newsagents. You know, I think there's a degree of vulnerability that's felt sometimes, by some of the smaller community organisations working and contributing towards the agenda.” - Local Authority1, Public Sector  “So people like the Community Trusts at a football organisation or cricket, or whichever sports providers, and then trying to sift through trying to find these pots of funding that are very compartmentalized in specific ward areas.” – Community Sport Organisation 2, Sport/PA Sector |
| *Monitoring and Evaluation of HP Projects* | “We're travelling over 3 sectors, you've got the health sector, you've got education sector, and you've got youth work and engagement sector. And therefore, we're having to talk three different languages to three different sorts of people. So it's really difficult to measure impact across that, and at no point am I saying sport there - it's just that sport is a theme throughout the three. So yeah, it's really challenging to be able to do that.” – PSCO4  “So XXX and I spent some time this afternoon looking at, yeah, Sport England delivery across the Southmead Hub and yeah, again I was blown away by the reach of the project, and the numbers of people that we managed to work with. But again, frustrated by the sort of lack of collaboration and joined up output and outcomes. So what we've done with other organisations, other partners, is really positive. It's just how we best demonstrate that as an output and an outcome really,” – PSCO3  “‘Okay, they didn't go to hospital, because they didn't self-harm. What did that cost?’ Again, that's very, very detailed stuff that we don't have the time to do at the moment, you know what I mean? When you, when you're a small organisation with staff that are out delivering all the time, going back to it, you know, we do need to do case studies.” – PSCO2  “I'm very, very confident it's all there. We just can't reach, we've got bits and pieces, you know. Our numbers are outstanding and so Easter holiday camps, for example, was 1735 children across 87 days of camp days, and they are great numbers…But that’s difficult one to shout about isn't, it doesn't really tell you a lot. It's like, ‘oh, yeah, you know, we're really good, and so people have stayed with us.’ That's not exactly a headline. – PSCO1  “It’s trying to draw out of those 8000, you know, so probably of those 8000, probably 7000 of those are receiving P.E lessons each week. What's the impact on those children through having really high quality P.E, as opposed to lower quality P.E?” – PSCO1 |
| **Demonstrating the impact of PSCOs** |  |
| *Unaware of current outputs* | “And whilst we would record those outputs (reach and engagement figures), [we] certainly wouldn't call them outcomes. And if you want to unlock significant money from the public sector, you've got to be a bit more sophisticated than that really.” – SC1, VCFSE Sector  “We are moving away though from just reporting hours, contact, as you said - those days have been and gone I think. And I think everybody now across the system has realised that doesn't tell you anything. We can make that data look like it's amazing. But actually, what's it really done?” – Active Partnership, Sport/PA Sector  “It’s still useful to have the stats of being like ‘80% of people who have [attended] improved their mental well-being’. But it is still, yes, that impact is the outcome of it, rather than just a X amount of kids came to a session.” – Community Sport Organisation 3, Sport/PA Sector  “It's about changing perceptions and the only way you can change perceptions is getting in front of people, building partnerships.” – PSCO4  “It's presenting it in the right format, to the right people, at the right time.” – Active Partnership, Sport/PA Sector  “So those Trusts coming to meetings and giving us the option of referring patients into those [programmes]. Or all coming in, and coming to meet some of our community organisations so patients [and] people can refer themselves into those activities. [That] would feel like a step in the right direction.” – PCN1, Health Sector  “The City Council and Sport England are putting a lot of investment in time, and resources, and money, and creating and fostering a stronger network - and Bristol Sport [Foundation], the Robins Foundation, Bristol Bears, were in the room, and so were Bristol Rovers - they're all part of the network. So I think that's what, we would be better as a group if we continued on this path for at the moment.” – Sport/PA Provider1, Private Sector  “So it's a someone within a Community Centre who enjoys, [and has] grown up in [that locality]…and it's finding that person who's generally connected with the Community Centre, who knows what the community [want], the value of the community, and what it means to people, and what the community can be - and they tend to be within the Community Centre.” – Community Sport Organisation 2, Sport/PA Sector |
| *How could M&E be improved?* | “I think it's a bit of both because we can be led by partners locally [or] nationally about what we need to do. But if we haven't still gotten the right people, persons, on the ground internally, then we're still just having a conversation about it, as opposed to being able to deliver it.” – PSCO3  “I think perhaps an organisation like [Active Partnership] would be somewhere you would go... I think we could probably go to them for help. I could probably ask the EFL. I think the EFL, if we wanted to, they would probably be able to sign post us to people that could help. I think the help is out there. I suppose again, it all comes down to the capacity.” – PSCO2  “The support services, or structures around [or]beyond the bread and butter of delivery, haven't kept pace… So I think that's going to be the next area of focus for the charity, is to make sure that we've got all those services around the delivery that make sure that we're able to supplement it with other funding that diversifies our income streams, makes us more safer as a going concern. [It] means that we can focus on communities that need it most as well, but quite clear with that sort of strategy moving forward. But then also shout about the work and demonstrate the work better through the social impact.” – PSCO1  “To actually do [that], we need that flexibility there, but to be able to just, you know, direct people in the right, right direction to go right, ‘if you're going to run these programmes, here's something that we would recommend as designed to do that’. You know, that that would be ideal.” – PSCO2    “Our youth engagement stuff is the stuff around reducing antisocial behaviour and knife crime and gangs and that stuff, [is that] in Sport England’s strategy? Probably not. Is there stuff around helping young people from areas of deprivation access proper education, in supporting the strategy? Probably not…It's really difficult to measure impact across that, and at no point am I saying sport there - it's just that sport is a theme throughout the three. So yeah, it's really challenging to be able to do that.” – PSCO4  “I think at the moment everything is, it's all quite an ad hoc sort of process, isn't it at the moment. But engaging, I think you said at the beginning, engaging the relevant stakeholders in terms of what that might look like to ensure that it's showing the right thing is going to be, it will be an important thing to take into account there.” –Local Authority1, Public Sector  “Yes, and probably even more broader than just the professional sports clubs. I think we need a common evaluation framework for all physical activity interventions, full stop... It might not be achievable. It might not because the nature of prevention is that it's so varied. So I don't, I really don't know if it is, but I think we can control what we can control and if there is a way of at least having commonality amongst professional sport club programmes then that's one thing. If we can achieve it across physical activity, that's another thing.” – Active Partnership, Sport/PA Sector  “I think it's something that we certainly would want to be involved in, for sure, because I think we would absolutely be able to add value in terms the national conversations going on around this– Active Partnership, Sport/PA Sector  “Well, it's difficult because there's no national [framework], you know, in terms of sport for change. There's no like, one accepted kind of impact measurement” - Community Sport Organisation1, Sport Sector  “It's [a common reporting framework] exactly, exactly what we need. Well, there will be challenges… But, I think everybody realises we've got to do something about inequality. Everybody realises the life-course. I think if we start it off at, you know, this is not about your building or how big your Saturday numbers are or whatever, then I think yeah, maybe it would work well.” – Local Authority2, Public Sector  “And this is something that we've discussed already with [a PSCO] and [a PSCO] in recent times…Is there one thing that we can collect that will demonstrate our collective reach? Yes, and that answer is yes. What it is - to be discussed and worked out. But if there was a level of steer from, yeah, those other organisations that give [could] us that sort of framework to work towards – PSCO3  “I see the value in an organisation being able to impactfully tell their story through impact reporting. But I also do see the need for the sector to be able to come together and go look as a sector we are making this difference, which is why you need to fund the sports sector…but I don't think you can just use national reporting for an individual organisation.” – PSCO4  “[It’s] apples and pears, you know, I think it would be great if, you know, the city had something like that [a common reporting framework], if Bristol had it, that we could all use. – PSCO2 |
|  | **Abbreviations**: PA: Physical Activity; PCN: Primary Care Network; PSCO: Professional Sports Clubs and Organisations |
